# Destructive *per continuitatem* spondylodiscitis after endovascular abdominal or thoracic aneurysm repair (EVAR/TEVAR): rare and untreatable?

**DOI:** 10.1007/s00402-020-03672-4

**Published:** 2020-11-18

**Authors:** Marc Dreimann, Yu-Mi Ryang, Benjamin Schoof, Darius Thiessen, Sven Oliver Eicker, Patrick Strube, Martin Stangenberg

**Affiliations:** 1 Department of Trauma and Orthopedic Surgery, University Hospital Hamburg Eppendorf, Martinistrasse 52, 20246 Hamburg, Germany; 2 Department of Neurosurgery, Helios Klinikum Berlin-Buch, Berlin, Germany; 3 Department of Neurosurgery, University Hospital Hamburg Eppendorf, Hamburg, Germany; 4 Department of Orthopaedic Surgery, Universitätsklinikum Jena, Campus Waldkliniken Eisenberg, Eisenberg, Germany

**Keywords:** EVAR, TEVAR, *Per continuitatem* spondylodiscitis, Spinal osteomyelitis

## Abstract

**Introduction:**

Very few publications have previously described spondylodiscitis as a potential complication of endovascular aortic procedures (EVAR/TEVAR). We present to our knowledge the first case series of spondylodiscitis following EVAR/TEVAR based on our data base. Particular focus was laid on the complexity of disease treatment and grave outcome perspectives from a spine surgeon’s point of view in this seriously affected patient group.

**Materials and methods:**

A retrospective analysis and chart review was performed for 11 out of 284 consecutive spondylodiscitis patients who underwent EVAR/TEVAR procedure and developed destructive *per continuitatem* spondylodiscitis.

**Results:**

All 11 patients had single or more level destructive spondylodiscitis adjacent to the thoracic/lumbar stent graft. In mean, four surgeries were performed per patient to treat this rare complication. Six out of eleven patients (55%) died within 6 months of first identification of *per continuitatem* spondylodiscitis. In four patients due to persisting infection of the graft and recurrence of the abscess formation, a persisting fistula from anterior approach to the skin was applied.

**Conclusions:**

Destructive *per continuitatem* spondylodiscitis is a rare and severe complication post-EVAR/TEVAR. Clinical and imaging features of anterior paravertebral disease and anterior vertebral body involvement suggest direct continuous spread of the graft infection to the adjacent vertebral column. The mortality rate of these severe infections is extremely high and treatment with a permanent fistula may be one salvage procedure.

## Introduction

In recent years, a modern minimally invasive procedure for aortic aneurysm treatment has been introduced, called endovascular aortic repair (EVAR). Since it was first described in 1991 [1], EVAR has become the preferred method over open surgical repair for the treatment of abdominal aortic aneurysms in patients with suitable anatomy due to its lower mortality rate and comparable long-term survival [2, 3]. Aortic graft infection is a well-known complication of EVAR; however, numerous studies have shown a very low incidence of 0.5–1% with a safe treatment by device explanation with in situ reconstruction [4, 5]. A retrospective study by Ducasse et al. noted an infection rate of 0.43% after 9739 endovascular procedures [6]. Comparable low infection rates have been described for the thoracic endovascular aortic repair (TEVAR) procedure, which is an adaption of the technique from infrarenal to thoracic aorta [7]. Despite the risk of infected aneurysms, EVAR has become a well-accepted treatment option in combination with long-term antibiotic therapy due to its lower early mortality rate [8].

Several studies have suggested predisposing risk factors that may be associated with infection of the stent graft following EVAR/TEVAR, including multiple endovascular/surgical procedures, an immunocompromised state and nosocomial blood stream septicemia [9, 10].

Spinal osteomyelitis, a potential complication following EVAR/TEVAR, has received little attention in the literature. To our knowledge, only one study and three case reports have been published to date [11–14].

Here, we present a case series of eleven patients who developed osteomyelitis following EVAR or TEVAR, paying particular attention to the outcome in this challenging group of patients.

## Materials and methods

A prospective chart documentation and retrospective analysis of all patients who underwent operative treatment due to vertebral osteomyelitis at two spine centres (centre Hamburg, period: July 2013-July 2019; centre Berlin, period: February 2018 - July 2019) was performed and matched to the EVAR/TEVAR patient cohorts. Statistics were computed using IBM SPSS v23.0, results were deemed statistically significant at *p* < 0.05. The study was conducted according to the local ethical standards of the city of Hamburg and the Declaration of Helsinki.

A total of 259 osteomyelitis patients were identified in centre Hamburg and 27 cases in centre Berlin, in total 286. The diagnosis of spondylodiscitis is based on clinical parameters, radiological changes, classical laboratory parameters as well as microbiological and histopathological examinations. Eleven patients (3.8%) met the clinical diagnosis criteria for operatively treated osteomyelitis following EVAR/TEVAR. Patient history was extracted from electronic health records, including vascular and vertebral imaging (computed tomography [CT] and magnetic resonance imaging [MRI]), clinical findings, microbiologic and histopathologic findings, as well as laboratory parameters including hemogram, infection parameters and blood culture.

The imaging criteria were the presence of endplate erosions, disc enhancement, disc abscess and the extent of paraspinal disease (presence of an epidural or paravertebral phlegmon/abscess and associated psoas involvement).

The antibiotic treatment plan was started intraoperatively mainly with double antibiotics (e.g. beta-lactam and fluoroquinolone antibiotics) and discussed weekly and adapted to the clinical situation within an interdisciplinary infection board (infectiology, microbiology, pathology and spine surgery). Duration of antibiotic treatment was adapted to clinical situation up to lifelong.

## Results

All 11 patients were male with an average age of 69 years (range 49–77 years). The main clinical features of the patients are outlined in Table 1. Eight patients underwent the EVAR procedure and three had TEVAR. Six patients had the procedure based on a mycotic aneurysm, while the remaining five patients were treated due to a non-inflammatory aortic aneurysm.

**Table 1:** Characteristics of all patients

Patient	Age (years)	EVAR/TEVAR	Indication	Time period index surgery to spinal surgery (m)	Number of surgeries	Death
1	69	EVAR	AAA	75	2	x, o.r
2	66	TEVAR	TAA	12	1	–
3	75	EVAR	AAA	12	8	–
4	77	EVAR	AAA	3	3	x
5	51	EVAR	m.AAA	1	4	–
6	49	TEVAR	m.TAA	0	8	x
7	77	EVAR	m.AAA	3	3	–
8	75	EVAR	m.AAA	2	5	x
9	77	EVAR	m.AAA	1	2	x
10	69	TEVAR	TAA	2	2	–
11	73	EVAR	m.AAA	0	4	x

*EVAR* endovascular aortic repair, *TEVAR* thoracic endovascular aortic repair, *AAA* abdominal aortic aneurysm, *TAA* thoracic aortic aneurysm, *m* mycotic, *(m)* months, *o.r.* death due to other reason, *X* patient died

**Fig. 1:**
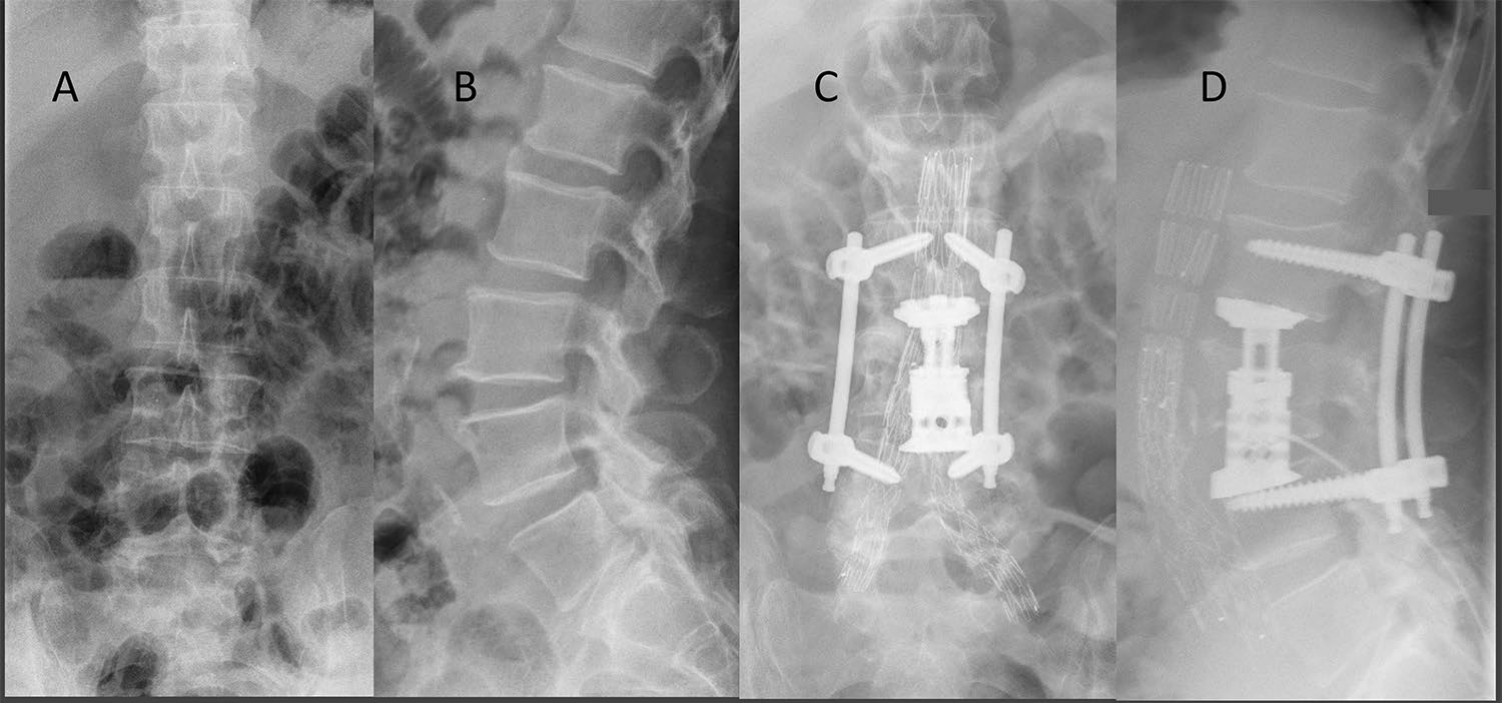
X-ray of patient 5 in the **a** anteroposterior and **b** lateral view at the beginning of clinical symptoms and after endovascular aortic repair (EVAR), posterior instrumentation and anterior vertebral body replacement

**Fig. 2:**
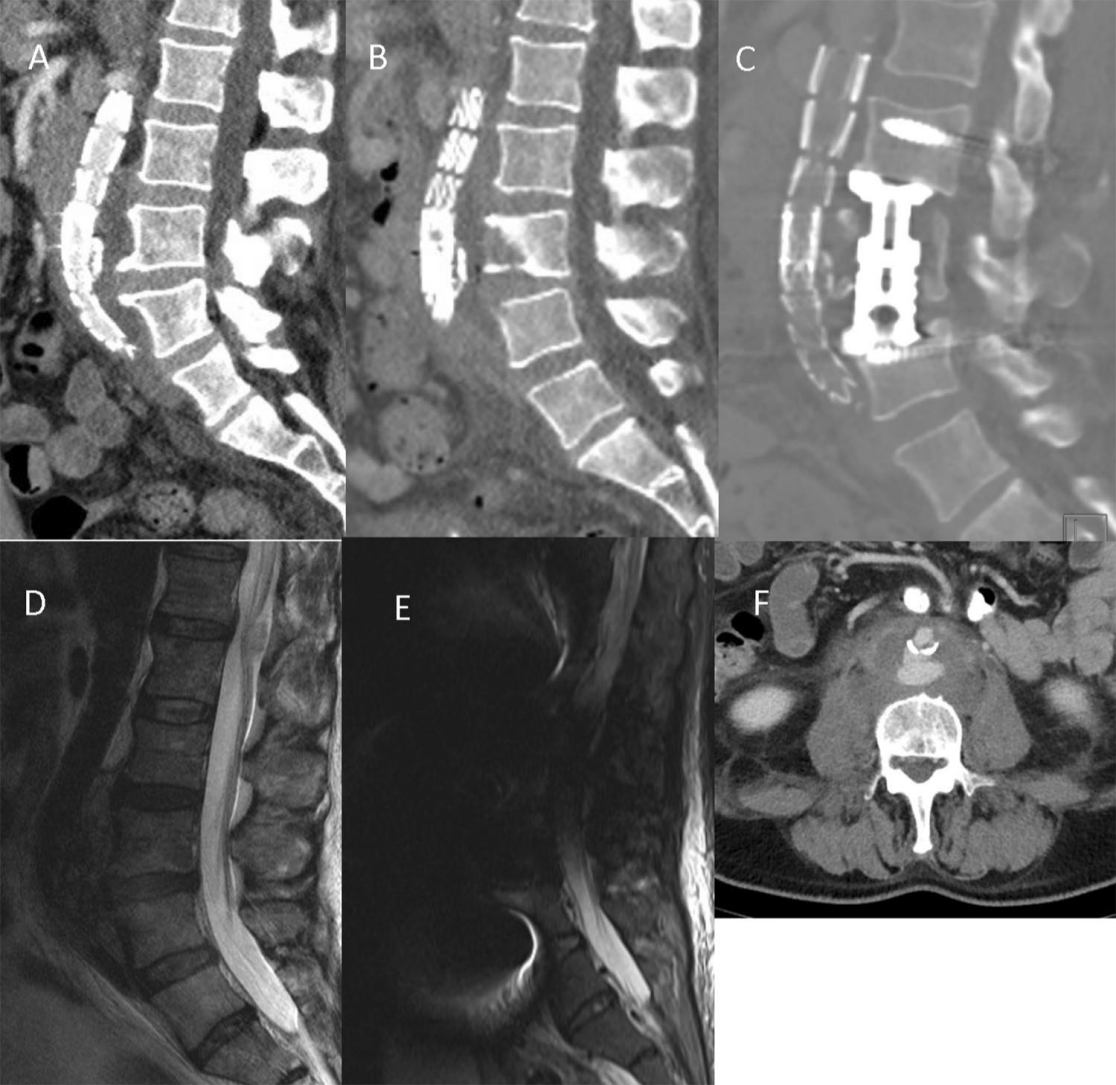
Computed tomography (CT) scan at **a** the beginning under antibiotic therapy and progressive anterior bony destruction and **b** after posterior instrumentation and anterior vertebral body replacement **c** in sagittal plane. Magnetic resonance imaging (MRI) at **d** the beginning of clinical symptoms and **e** after spinal surgery. **f** Axial CT scan with perifocal abscess around endovascular aortic repair (EVAR)

The clinical presentation following EVAR/TEVAR and identification of spondylodiscitis were variable. Three patients presented in the outpatient clinic with back pain after a period of 1 year or longer after the endovascular procedure. Eight patients presented through the vascular surgery department in septic or early septic conditions a maximum of 3 months after the procedure. The mean preoperative C-reactive protein (CRP) level was 143 mg/L (range 5–322 mg/L, *n* = < 5). An average of four surgeries (range 1–8, Table 2, Figs. 1 and 2) was performed to treat the spinal osteomyelitis.

**Table 2:** Treatments applied during surgeries

Patient	1	2	3	4	5	6	7	8
1	I: L1-4 D: L2-3, RP	WR p						
2	I: T3-6 D: T4-5							
3	I: L1-5 RP	IR	WR p	WR p F	WR p F	WR p	WR p F	WR p F
4	I: L2-S1 D: L4-S1	R EVAR, WR a	WR a					
5	I: L2-4 D: L2-4, VBR L3	WR a	WR a VBR R F	WR p F				
6	I: T 2-8 D: T3-6	WR p	VAC p	VAC p	I R	VAC	WR p F	WR p F
7	R EVAR VBR L4	I L2-S1	VBR R					
8	I: L3-5 D: L3-4	R EVAR VBR L4	WR a	I: L2-5	R EVAR R VBR			
9	I: Th10-L2 VBR Th12	R VBR Pleurectomy						
10	I: T 3–8 D: T5-6 WBR 5–6	WR p						
11	I: t12-L5 D: L2-4	VBR: L3	VAC	F				

*I* instrumentation, *D* decompression, *RP* revision abscess of psoas muscle, *VBR* vertebral body replacement. Revision endovascular aortic repair (EVAR). *WR* wound revision, *p* posterior, *a* anterior, *IR* implant removal, *f* fistula, *VAC* vacuum-assisted closure

In four patients, the treatment goal of primary requested complete infection restoration failed and a permanent spinal to cutaneous fistula was applied to avoid a septic relapse. In this situation, an abnormal passageway was created using a tube from the infected area of the anterior spine/perigraft to the skin.

Positive microbiological cultures were identified in eight out of eleven patients (73%), (*Staphylococcus aureus* in three patients, *Staphylococcus epidermidis* in two patients and *Peptoniphilus harei*, *Escherichia coli* and *Clostridium septucum* in one patient, respectively). All eight patients obtained positive biopsies from paravertebral samples collected during surgery. Two patients had corresponding positive blood cultures.

Significant comorbidities included hypertension in eight (72%), pulmonary disease in five (45%), ischemic heart disease in four (36%), diabetes in two (18%) and prior malignancy in three (27%) patients, and one patient suffered from alcohol and intravenous drug abuse, HIV and hepatitis B infection.

### Radiographic data

The key imaging features of osteomyelitis, identified in all eleven patients, are outlined in Table 3. Five patients presented with involvement of a single motion segment (3× lumbar, 2× thoracic), five patients with involvement of two motion segments (4× lumbar, 1× thoracic), and one patient showed involvement of three vertebral motion segments after TEVAR treatment. All levels of spinal osteomyelitis were adjacent to the vascular stent graft; they were classified as *per continuitatem* osteomyelitis.

**Table 3:** Infected level

Patient	Infected level	Number of levels	Psoas muscle abscess	Epidural abscess	Intraoperative biopsy	Preoperative antibiotic treatment	Preoperative blood culture	Septicaemia
1	L2/3	1	y	N	pos	n	neg	y
2	T4/5	1	–	N	neg	y	neg	y
3	L3/4	1	y	N	neg	y	neg	n
4	L4/S1	2	y	Y	pos	y	pos	n
5	L2/4	2	y	N	pos	y	neg	y
6	T3/6	3	–	Y	pos	y	pos	y
7	L3/5	2	y	N	pos	y	neg	y
8	L3/4	1	y	N	pos	y	neg	n
9	T11/L1	2	–	N	pos	y	neg	y
10	T5/6	1	–	N	pos	y	neg	n
11	L2/4	2	y	n	neg	y	neg	y

*L* lumbar, *T* thoracic. Number of levels that spondylodiscitis can be detected. *N* no, *y* yes, *pos* positive, *neg* negative

In some cases, an X-ray was performed, as shown in Fig. 1, but osteomyelitis-specific features were identified on CT, demonstrating endplate erosion (11/11) with or without anterior vertebral body erosion (10/11), loss of disc height, intervertebral disc enhancement (11/11) and paravertebral soft tissue/abscess (Fig. 2). All patients underwent at least one spinal MRI with limited image quality due to extensive susceptibility artefacts arising from the adjacent stent graft. A higher sensitivity for intraspinal abscesses was the indication for spinal MRI, which could be detected in two out of nine patients in terms of epidural abscess formation without neurological deterioration.

**Fig. 3:**
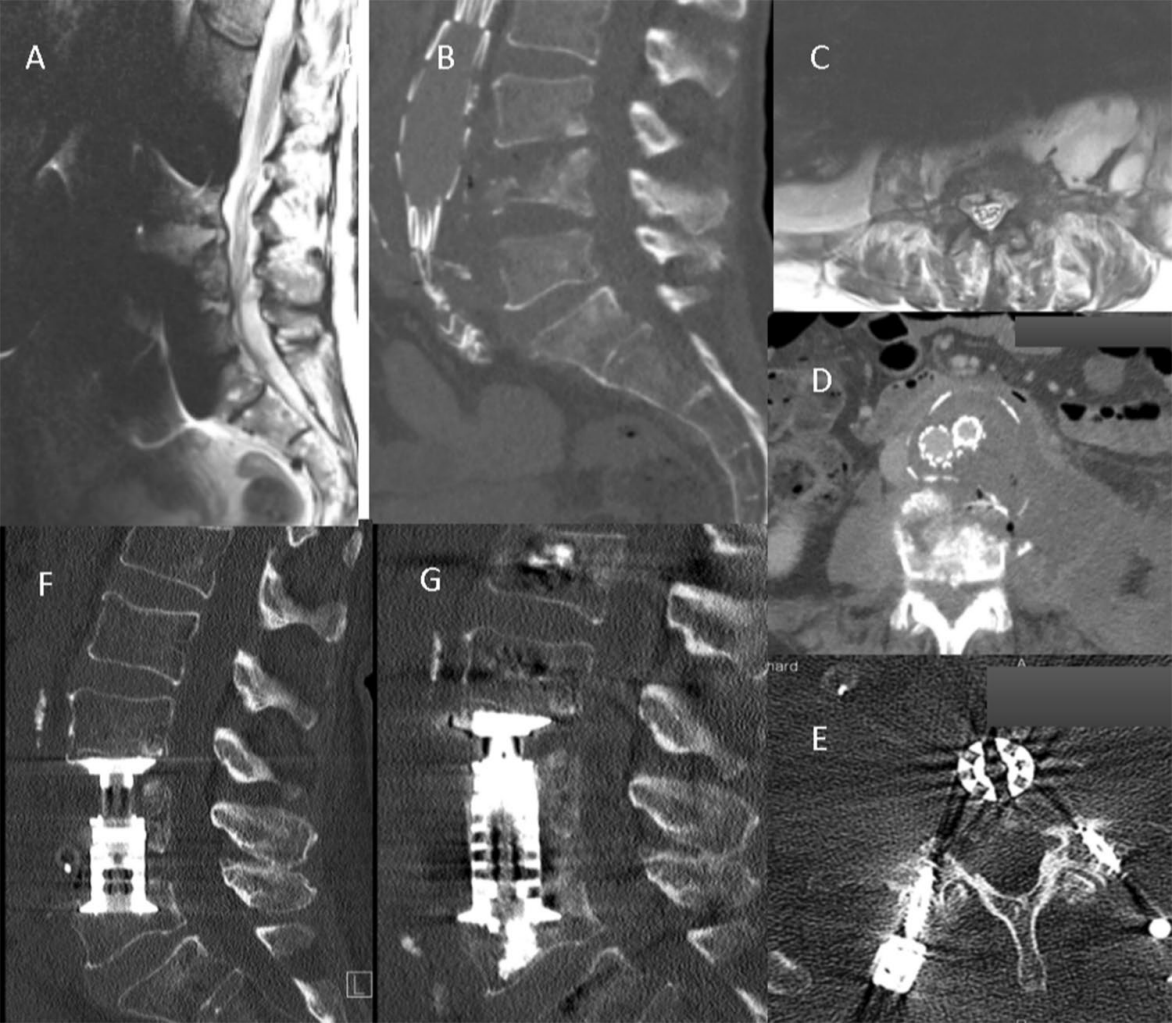
**a, c** Magnetic resonance imaging (MRI) with endovascular aortic repair (EVAR) in place and large abscesses. **b, d** Vertebral body destruction with *per continuitatem* infection to the vessel prothesis. **f** Computed tomography (CT) scan showing the loss of height of L5 with revision surgery from posterior in (**e**, **g**)

All seven patients with lumbar spondylodiscitis had paravertebral abscesses of the psoas muscle (Fig. 3) or phlegmon of varying sizes, which were either contiguous with or immediately adjacent to the native aneurysmal aortic sac. All TEVAR patients also showed inflammation adjacent to the thoracic aorta. The mycotic aneurysms are described as a mushroom-shaped aneurysm. This may create considerable confusion, since "mycotic" is typically used to define fungal infections. However, mycotic aneurysm is still used for all aneurysms caused by infections, except for syphilitic aortitis.

## Treatment, progress and complications

All patients received antibiotic treatment after interdisciplinary microbiological team decisions. The interdisciplinary team comprised of infectiology experts, internal medicine experts, virologists, microbiologists, pathologists and spine surgeons.

Six out of eleven patients (55%) died within 6 months of being diagnosed with osteomyelitis (one patient died secondary to vascular complications of graft revision, one patient had advanced recurrent prostate malignancy, and four patients had progressive inflammation/sepsis).

Of the five out of eleven patients who survived, only one underwent removal of the stent graft with aortic reconstruction, vertebral debridement, decompression and spinal fusion (Fig. 3). The remaining four patients were successfully treated for osteomyelitis with vertebral debridement, decompression, spinal fusion and antibiotic treatment. Due to persisting infection of the graft and recurrence of abscess formation without spinal progression, a persisting fistula from the anterior approach to the skin was applied. In all four patients, life-long antibiotic treatment was prescribed. The antibiotic procedure was discussed with the interdisciplinary infection board and adapted to the detected microbiological organism.

## Discussion

Since the initial reports of endovascular stent grafts (EVAR, TEVAR), their use has increased dramatically in both the infrarenal and thoracic aorta [1]. Endograft infection may affect approximately 1% of all implantations [5, 15]; however, the available evidence is of low quality.

A systematic review identified 117 published cases (34 thoracic, 83 abdominal), mostly case reports, which compared different management options [6]. Traditionally, endograft infection has been managed by debridement of infected tissue and arterial reconstruction, followed by prolonged antibiotic therapy [16]. The outcome of infected abdominal and thoracic endografts is poor, with estimated overall short- to medium-term survival rates of 65% and 30%, respectively [16].

Spondylodiscitis is the most common spinal infection. It affects the intervertebral disk, adjacent vertebral bodies, and occasionally also the posterior elements of the spine [17]. The incidence of spondylodiscitis has increased over the last 2 decades to 30 per 250,000 individuals annually in Western countries [18]. This increase in incidence is thought to be due to the aging population, the rise in immunosuppressed patients, intravenous drug use and improved diagnostic availability [18, 19].

Although diagnostic and therapeutic options have drastically improved over the past decades, spinal osteomyelitis remains a diagnostic and therapeutic challenge, with a mortality rate of 2–20% [20]. In combination with an infected EVAR/TEVAR as the origin of per continuitatem spondylodiscitis, the mortality rate rises to 55%.

Subsequent ischemia and disc necrosis can arise with the development of osteomyelitis, as observed in this special patient cohort. By this, the correlating level of the spinal and vascular infection becomes explainable. From this point of view, osteomyelitis might be the focus of infection. Osteomyelitis may also pre-exist and perfusion is limited by the endograft. However, the impact of TEVAR and EVAR on spinal cord ischemia is well established and is based on perfusion of the same collateral network that supplies the spine [21]. The pattern of extensive anterior vertebral bony destruction and paravertebral abscess formation next to the endovascularly treated aortic aneurysm suggests direct spread from the adjacent infected aortic stent graft and native aneurysm sac as a likely route of *per continuitatem* vertebral column infection, resulting in a double infection of the graft and the vertebral body. Due to the endovascular nature of the stent a haematogenous spread of pathogenic agents might also be a possible route of infection.

These findings highlight the observation that patients with EVAR/TEVAR develop a more ventrally located osteomyelitis with extended prevertebral involvement in terms of infection of the stent or disc due to the decreased vascular ventral blood supply. This may also explain why only two of the eleven (18%) patients in this cohort presented disease extension to the epidural space (Table 3).

Mandegaran et al. [14] presented the first case series of osteomyelitis after EVAR. According to these authors, the extended anterior paravertebral infection and the anterior vertebral body bony involvement suggest direct spread of the infection from the infected graft to the adjacent vertebral column.

The presence of an already infected aneurysm and pre-existing septicaemia before any vascular intervention has been shown to be strongly associated with the development of osteomyelitis next to the graft [22]. This is in line with the acute development of osteomyelitis of mycotic aneurysms (range 0–3 months) which is the infection and subsequent severe destruction of the vertebral column (Fig. 3).

It is notable that although all eleven patients in our study demonstrated the clinical features of spondylodiscitis, obtained blood culture were only positive in two patients. The explanation for this might be the high rate of preoperative antibiotic treatment (ten out of eleven patients, 91%), which might decrease the yield for positive blood cultures. Intraoperative biopsies were positive in eight out of eleven samples (73%), and out of the two patients with no causative organism identified, one had a positive histological biopsy for bacteria. This is in line with previous studies that have highlighted the limited sensitivity of a paravertebral phlegmon/abscess biopsies (68%) [23]. The diagnosis of vertebral osteomyelitis, therefore, usually is made from a combination of radiological, clinical, laboratory and microbiological results [18].

With regard to the imaging assessment of vertebral osteomyelitis following aortic procedures, our retrospective cohort of patients underwent varying combinations of CT and MRI imaging since extended artefacts pose a challenge to obtaining imaging sustaining the suspected diagnosis. Across these modalities, the typical features of pyogenic osteomyelitis were identified in all eleven patients, erosion/destruction of adjacent vertebral endplates, signal abnormality and enhanced uptake of contrast agent of the involved disc(s) and adjacent endplates and paravertebral abscess formations [24].

CT imaging is able to depict morphological changes in the native aneurysm sac compared to preoperative studies (Fig. 3), in addition to new aortic rim enhancement and internal gas, indicating infection of the stent graft and aneurysm sac. However, assessment of epidural involvement and suspected paravertebral abscess was challenging. Therefore, gadolinium-enhanced MRI is considered the gold standard in the assessment of osteomyelitis [24].

A study by Ducasse [6] described the best outcome after surgical excision of the infected stent graft followed by in situ reconstruction. This seems to be the best treating modality of isolated aorto-iliac stent graft infections. In this study, treatment was surgical in 82% of cases and was performed by stent graft removal and followed by either extra-anatomical bypass (60%) or in situ prosthetic reconstruction (40%). Mortality was 18% overall: 36.4% after conservative treatment and 14% after surgical treatment. In our group, the total revision with graft change and in situ reconstruction was followed by the death of two patients who had developed sepsis.

The subgroup of mycotic aneurysms has a high mortality even in open or endovascular technique. Antibiotic treatment for more than 6 months post-operatively was associated with better survival [25]. However, MAA treatment should always be tailor made and planned individually, and general recommendations are in vain. Same is for the small number of patients with *per continuitatem* spondylodiscitis, either mycotic or non-mycotic.

**Fig. 4:**
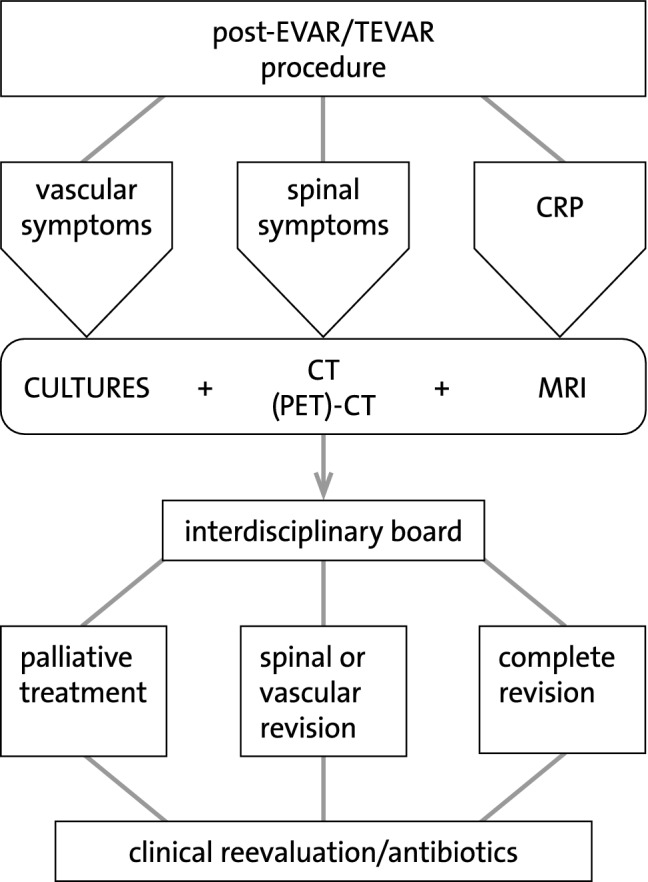
Proposed algorithm for workup and management of patients with infected endograft and *per continuitatem* spondylodiscitis

It seems that the creation of the persistent fistula enabled the survival of these patients. Further treatment plans like primary vacuum-assisted closure (VAC) therapy may improve the results of this difficult group of patients. This is addressed in the flowchart of Fig. 4.

## Limitations

Our trial has several limitations. First, its retrospective nature and the short follow-up period. Consequently, the small sample size, lack of validated outcome scores and patient selection might have contributed to a certain selection bias. Second, our trial was conducted in two centers, which limits its generalizability. Third, a non-homogenous group of both mycotic and non-mycotic AAAs was put together.

## Conclusions

From these results we conclude, that aortic stent graft infection with destructive vertebral *per continuitatem* osteomyelitis is an uncommon occurrence with low evidence. Surgical treatment is difficult and complex. Revision and permanent fistula seem to provide an acceptable outcome in this patient group at high risk. Further studies are needed to identify patients at greatest risk for infection of EVAR/TEVAR, which would necessitate enhanced surveillance, longer treatment of intervening infections, or prophylactic antibiotic treatment to preserve the endograft. This will be difficult to accomplish due to the low incidence of infected EVARs/TEVARs.
